# Vascular survey of the maxillary vestibule and gingiva—clinical impact on incision and flap design in periodontal and implant surgeries

**DOI:** 10.1007/s00784-020-03419-w

**Published:** 2020-07-07

**Authors:** Arvin Shahbazi, Georg Feigl, Anton Sculean, András Grimm, Dániel Palkovics, Bálint Molnár, Péter Windisch

**Affiliations:** 1 Department of Anatomy, Histology and Embryology, Faculty of Medicine, Semmelweis University, Budapest, Hungary; 2 Department of Periodontology, Faculty of Dentistry, Semmelweis University, Budapest, Hungary; 3 Department of Macroscopical and Clinical Anatomy, Medical University of Graz, Graz, Austria; 4 Department of Periodontology, School of Dental Medicine, University of Bern, Bern, Switzerland; 5 Department of Otolaryngology and Head and Neck Surgery, Semmelweis University, Budapest, Hungary

**Keywords:** Dental implant, Incision design, Split thickness flap, Angiogenesis, Wound healing

## Abstract

**Objectives:**

Currently, empirical clinical findings on the blood supply of the maxillary vestibule are not backed up with sufficient literature. The purpose of this study was to investigate the mucosal and periosteal vascular supply in the maxillary vestibule macroscopically and radiographically to improve surgical strategies and flap designs.

**Materials and methods:**

Thirty head corpses were selected (9 dentate, 11 partially edentulous, 10 edentulous). Twenty-six corpses were injected by red latex milk and embalmed with Thiel solution. Four cadavers were prepared for corrosion casting. Arterial path and anastomoses in the maxillary vestibule of dentate, partially edentulous and edentulous ridges, were analyzed macroscopically and by computed tomography (CT).

**Results:**

Transverse periosteomucosal anastomoses were detected in the posterior and esthetic zones of the maxillary vestibule. The buccal branches penetrated the interdental septum toward the palate. In the esthetic zone, superior labial artery (SLA) supplied the mucosa and the infraorbital artery (IOA) supplied the periosteum. Corrosion casting showed anastomoses between IOA and nasal septal branches. CT analysis revealed ipsilateral and contralateral anastomoses between SLA and IOA. In dentate ridges, mucosal star-shaped terminal branches were detected.

**Conclusions:**

The macroscopic and radiographic vascular survey analysis revealed the anatomical background behind several clinically documented phenomena related to oral and periodontal surgeries.

**Clinical relevance:**

This study permits clinicians to design less invasive flaps when releasing incisions in the maxillary vestibule during periodontal and implant surgeries. Our observations strongly point to the significance of an undamaged periosteum to prevent compromised flap revascularization and wound healing disturbances.

## Introduction

In implant-related dentoalveolar and periodontal surgeries, the comprehensive oral topographical knowledge is significant for incision design [1]. To avert complications in neovascularization [2] and wound healing [3–5], detailed erudition about the route and branching of adjacent mucosal, muscular, and periosteal vessels is required. Amid all oral surgical fields, the vestibule of the upper jaw, particularly the anterior maxilla, demands the most sophisticated and foreseen surgical care due to possible esthetic disturbances provoked by impaired blood supply [6–9].

The vascular path and morphology of the maxillary vestibule have an essential impact on the outcome of reconstructive surgical interventions by influencing intraoperative bleeding and postoperative revascularization [10, 11]. The upper oral vestibule is chiefly furnished by the branches of maxillary and facial arteries. The maxillary artery (MA) supplies the bony maxilla, maxillary sinus, upper teeth, gingiva and hard palate by the posterior superior alveolar artery (PSAA), the infraorbital artery (IOA), the greater palatine artery (GPA), and the nasopalatine artery (NPA) [12–15]. The facial artery (FA) gives the superior labial artery (SLA) at the level of the labial angle. The SLA runs medially and anastomoses with the contralateral SLA and forms the arterial circle around the mouth together with the inferior labial arteries (ILA) [16]. The complex arterial circuit with affluence of collateral sources of blood flow is crucial when surgical flaps are designed in the anterior vestibule.

The immense bulk of surgical implications such as implantation, sinus floor elevation, and ridge augmentation in the maxillary vestibule is based on full-thickness mucoperiosteal flap designs with unilateral or bilateral full-thickness vertical and horizontal periosteal releasing incisions [17]. The periosteal releasing incision is described for a tension-free flap design and significant coronal flap advancement [18]. Even though these treatments performed by skilled surgeons result in a successful outcome, still there are intra- and postoperative complications linked to extensive flap mobilization such as increased intraoperative bleeding, disturbance of mucosal, periosteal neurovascular supply and wound healing, partial flap necrosis, and vertical scars [19, 20]. Furthermore, distortion of the vestibule and loss of keratinized mucosa over the edentulous ridge are frequently observed. These sequels may damage the ultimate esthetic and functional result of surgeries in the upper vestibule.

Several authors described the split-thickness flap in implant dentistry, which might represent a less compromised postoperative flap circulation because of the scarcity of vertical or horizontal periosteal releasing incisions, thereby protecting collateral blood vessels. In 2002, Cranin explained various flap strategies for soft tissue management in implant surgery including the split-thickness approach to promote esthetic and functional outcomes of peri-implant soft tissues [21]. To accomplish success with guided bone regeneration (GBR), tensionless flap closure is a critical factor. Greenstein et al. in 2009 reviewed numerous flap designs to determine the proper method which can contribute to tensionless primary closure with the least amount of morbidity [22]. They reported that many crucial factors such as tissue elasticity, size of the vestibule, size of augmentation, oral musculature, and operator expertise might determine the outcomes of GBR [22]. In 2012, Steigmann et al. concluded that splitting the mucoperiosteal flap can enhance soft tissue mobility and elasticity significantly and cover severe ridge paucities when utilizing the periosteal pocket for GBR [23]. In 2010, Hur et al. and then Ogata et al. (2013) proposed a split-thickness flap approach for ridge augmentation with a single mesial split-thickness vertical incision [24, 25]. Windisch and co-workers (2017) introduced a split-thickness flap design for GBR without vertical releasing incisions followed by periosteal-mucosal two-layer flap closure [26]. The authors have observed a low number of postoperative complications with undisturbed wound healing. Nevertheless, accurate anatomical data are lacking in the literature, which might restrict clinicians to understand blood supply disruptions associated with different mucosal/periosteal incision techniques. Consequently, establishing an evidence-based anatomical guideline by proper outlining of the vascular course for planning surgical interventions in the upper vestibule is crucial.

## Aims

The goals of this study were (i) to macroscopically analyze the path of the arteries in the maxillary vestibule by using latex milk injection and corrosion casting, and (ii) to perform a radiographic investigation of vascular survey and distribution by computed tomography (CT) by using latex milk injection.

We aimed at mapping the mucosal and periosteal blood supply as well as collateral anastomoses to establish an anatomical foundation to improve current surgical strategies utilizing full- and split-thickness flap designs in the field of implant dentistry and periodontology.

## Materials and methods

Thirty head corpses were selected for this study (9 dentate, 11 partially edentulous, 10 edentulous). All preparations were performed by trained investigators (A.S, G.F, A.G).

Twenty-six cadavers (14 males, 12 females; 60–95 years of age) were injected with Thiel’s solution and latex milk followed by a macroscopic and radiographic vascular examination. The cadavers were donated to the Department of Macroscopic and Clinical Anatomy of the Medical University of Graz, Austria, according to the Department’s donation program and the Styrian burial law.

Four cadavers (2 males, 2 females; 64–73 years of age) were prepared for corrosion casting macroscopical analysis. Specimens were donated to the Department of Anatomy, Histology and Embryology, Semmelweis University, Budapest, Hungary, for education and research proposals according to the Hungarian approval rules of anatomical donation.

## Latex milk injection

The method of injection was followed according to Shahbazi et al. [27]. The common carotid arteries (CCAs) were flushed with Thiel solution [28–32] close to pressure of 0.5 bar. Then, 20–30 ml of diluted ammonia was injected to evacuate the vessels and withdraw coagulation*.* Subsequently, the latex milk (Creato Latexmilch, Zitzmann Zentrale, Baden, Germany) mixed with a red color agent (Pintasol red E-L3 mix paste, Mixol-products Diebold GmbH, Kirchheim, Germany) was injected through the CCA. The cadavers were fixed within Thiel solution [28–32] for about 6–8 months. After termination of the embalming period, the corpses were sealed in zipper polyethylene plastic bags for another 6 months with chlorocresol. Following the fixation period, injected specimens were dissected by Nr. 15C surgical blades under × 2.5 magnification. The mucosa was separated from the periosteum, and stained arteries were detected. The route of the vessels with their anastomoses was macroscopically studied.

## Corrosion casting

After dissection of external carotid arteries (ECAs), they were washed by the phosphate buffer saline solution. In the casting phase, the acrylic resin (ACRIFIX 190 (2 R 0190), Evonik Industries AG., Germany), which included a red color agent (AKEMI GmbH., Nurnberg, Germany), was injected into the ECAs. During this phase, the head corpses were retained in a stable position, depending on individual conditions [33, 34]. Then, the specimens were kept in water at a temperature intermitting between 40 and 60 ° C for 24 h for full polymerization of the resin [33, 34]. In the corrosion phase, the tissues around polymerized resin were macerated by Somat gold 12 actions (Henkel AG., Germany) dishwasher tabs at 36 °C temperature for about 2 months. During the corrosion phase, the solution was changed several times to eliminate debris and to enhance the visualization of the structures of interest [35].

## Radiological analysis

### Computed tomography

Before dissection, the latex milk–injected head specimens were analyzed by multislice, spiral CT (Siemens, Somatom Emotion, Munich, Germany). Since the latex milk is radiopaque [27], vascular surveys of the MA, PSAA, IOA, FA, and SLA were investigated. Their sub-branches in the maxillary vestibule together with their anastomoses and divisions were compared.

## Results

**Fig. 1:**
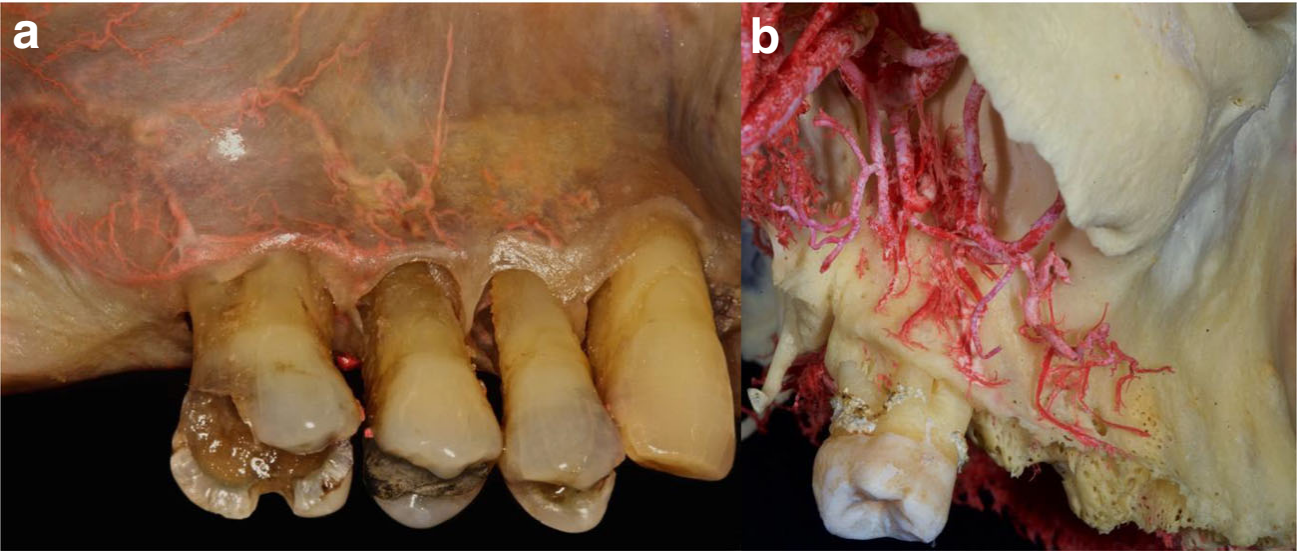
Horizontal course of the posterior superior alveolar artery (PSAA) with the sub-branches. **a** Latex milk injection. **b** Corrosion casting

**Fig. 2:**
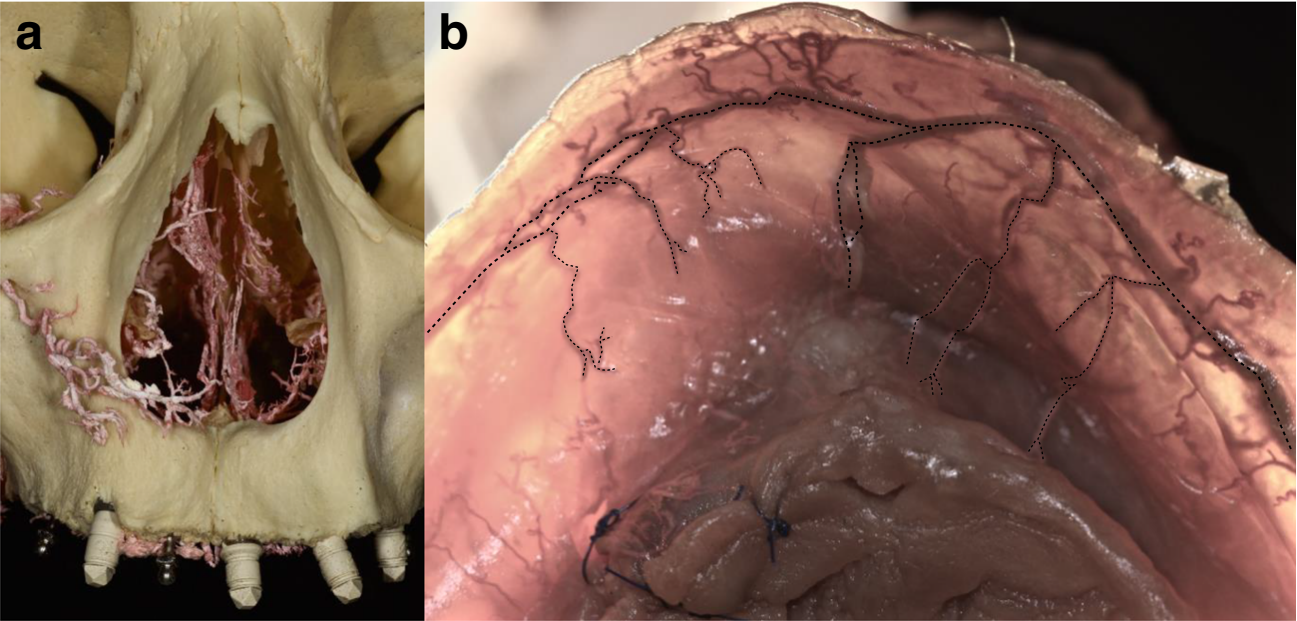
Anastomoses in the anterior maxillary vestibule. **a** Infraorbital artery (IOA) anastomoses with the septal branches of the nasal cavity (corrosion casting). **b** Anastomoses between the superior labial arteries (SLA), together with the vertical mucosal branches (latex milk injection)

**Fig. 3:**
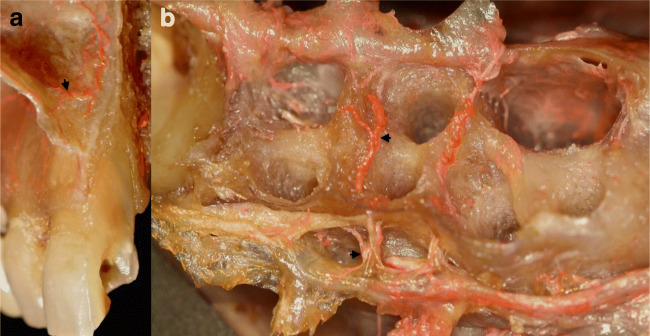
Transverse anastomoses between the mucosa and periosteum (latex milk injection). **a** Anastomoses between the vertical branches of the mucosa deriving from superior labial artery (SLA) and vertical branches deriving from the infraorbital artery (IOA). **b** Transverse anastomosis between the mucosa and periosteum, penetrating the buccal bone, passing on the inter-alveolar septum, and piercing the palatal bone to connect with the greater palatine artery (GPA)

**Fig. 4:**
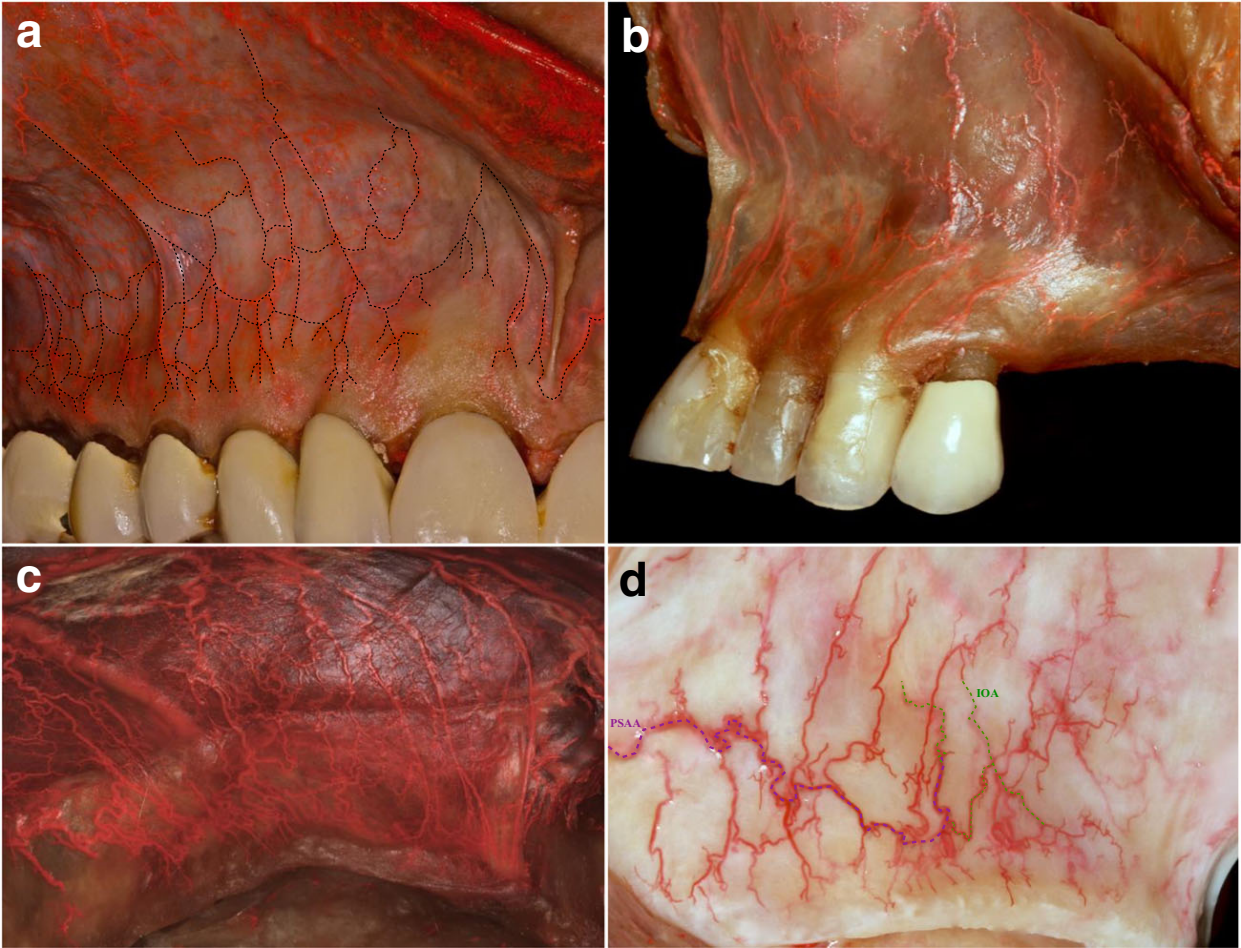
Mucosal vascular survey of the maxillary vestibule in dentate, partially dentate, and edentulous ridges (latex milk injection). **a** Vertical mucosal branches bifurcating at the level of movable and attached mucosa forming a loop anastomoses; from each loop, 4–5 vertical branches supply the gingival limbus. **b** Vascular supply of the anterior vestibule, with the vertical branches moving to distal and mesial sides of the papillae. **c** Vestibular supply of the edentulous anterior maxilla. Due to bone and connective tissue resorption, the mucosal and periosteal vertical branches show irregular and enlarged pattern, even the superior labial artery (SLA) is visible. **d** Vestibular supply of the edentulous posterior maxilla. Horizontal, curvy pattern of posterior superior alveolar artery (PSAA) is observable, anastomosing with the deep vertical infraorbital artery (IOA)

**Fig. 5:**
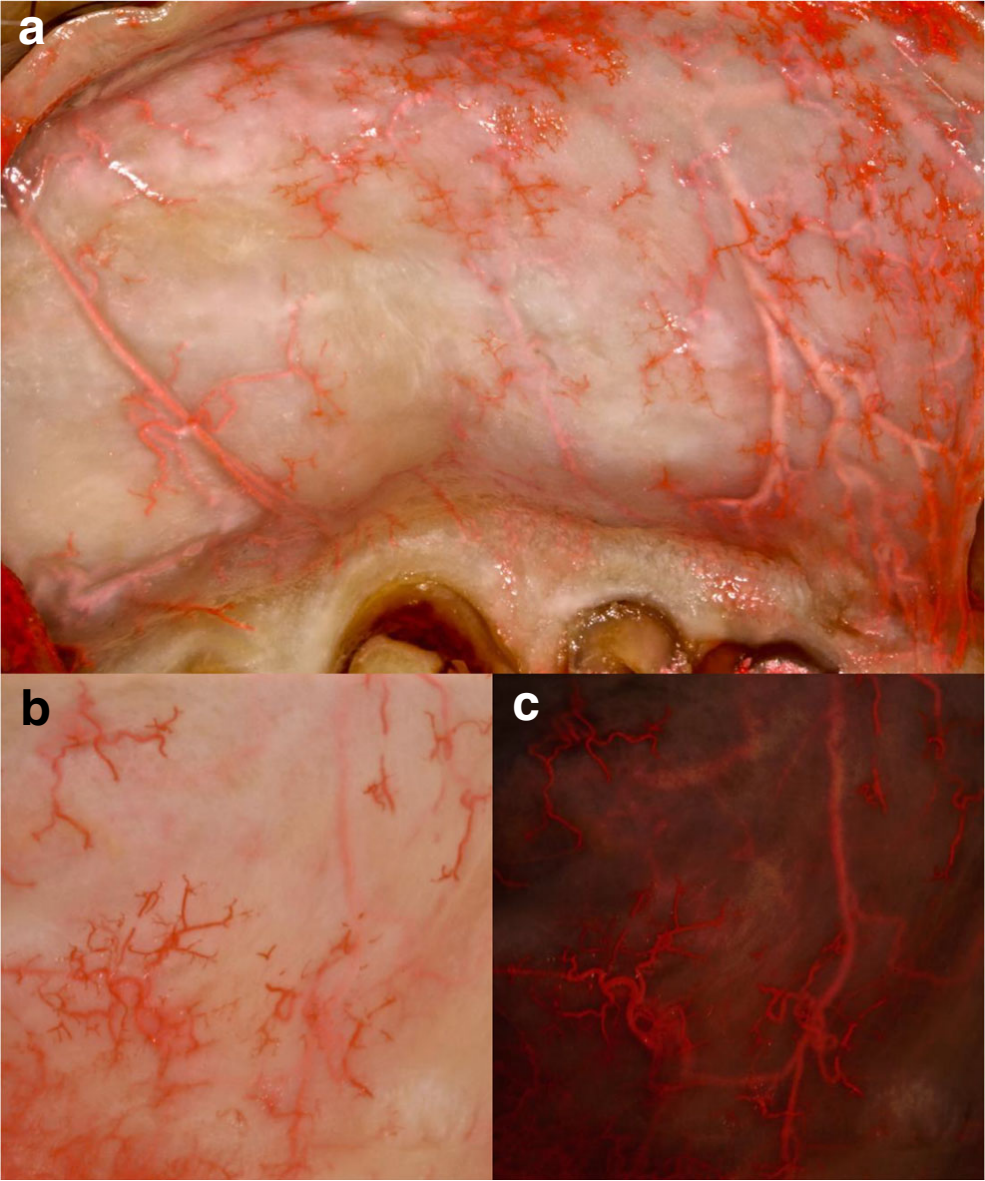
Superior labial artery (SLA) with the vertical and terminal (star-shaped) branches supplying the esthetic zone mucosa and upper labial frenulum (latex milk injection). **a** Overview of the vertical branches of the SLA, forming an anastomoses, supplying the labial frenulum, gingiva and giving mucosal terminal (star-shaped) branches. **b** Magnified star-shaped terminal branches. **c** The submucosal artery giving the terminal branches to the mucosa

**Fig. 6:**
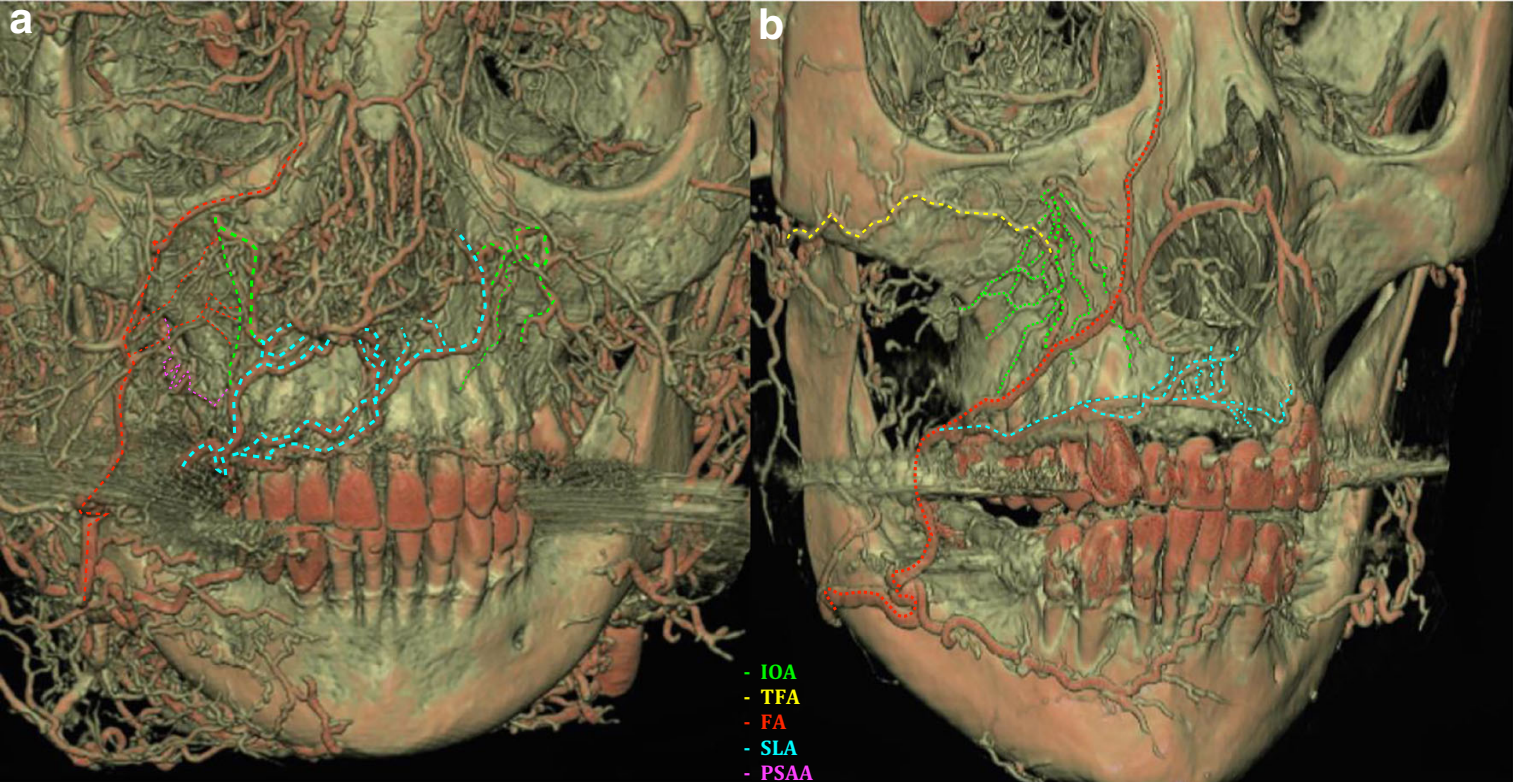
Computed tomography (CT) analysis vascular survey injected by red latex milk. **a** Anterior view of the face, anastomoses among the sub-branches of the facial artery (FA) and infraorbital artery (IOA), posterior superior alveolar artery (PSAA), and IOA are marked; also, the superior labial artery (SLA) forms anastomoses with the nasal septal, ipsi- and contralateral IOA branches. **b** Several anastomoses among IOA and transverse facial artery (TFA), IOA, and FA are labeled

The routes of PSAA, IOA, FA, and SLA with their sub-branches and anastomoses in the maxillary vestibule of dentate, partially edentulous and edentulous ridges, were analyzed macroscopically (Figs. 1, 2, 3, 4, 5) and radiographically (Fig. 6). PSAA with its transverse course chiefly supplied the mucosa and the periosteum of the posterior portion of the maxillary vestibule (Fig. 1). However, the mucosa and the periosteum of the upper premolars were supplied by PSAA and IOA. At this level, an anastomosis between the PSAA and IOA was macroscopically and radiographically identified (Figs. 4, 6).

In the esthetic zone, the SLA supplied the mucosa; nevertheless, the periosteal layer of this zone was supplied by vertical branches of the IOA (Figs. 2, 4, 5). By the corrosion casting method, we were able to detect anastomoses between the IOA and nasal septal branches of the nasal cavity (Fig. 2). Furthermore, by CT analysis, ipsilateral and contralateral anastomoses between SLA and IOA were observed (Fig. 6). Numerous vessels such as FA and the transverse facial artery formed complex anastomoses with IOA (Fig. 6). In the mucosa of the dentate preparations, coronal to the mucogingival junction, the vertical branches emanating from PSAA, IOA, and SLA bifurcated in an organized manner and created an arterial loop anastomosis (Fig. 4). From these loops, around 4 to 5 vertically oriented branches supplied the marginal gingiva and the papillae. Moreover, many direct vertical branches toward the papillae were observed (Fig. 4). The differences in the course of blood supply of maxillary vestibule between dentate and edentulous ridges were compared. In the mucosa of the dentate specimens, a regular loop pattern of arterial distribution was observed. However, in non-dentate ridges, due to resorption of the connective tissue, the vessels appeared to have an irregular, undulating course and they were enlarged (Fig. 4).

**Fig. 7:**
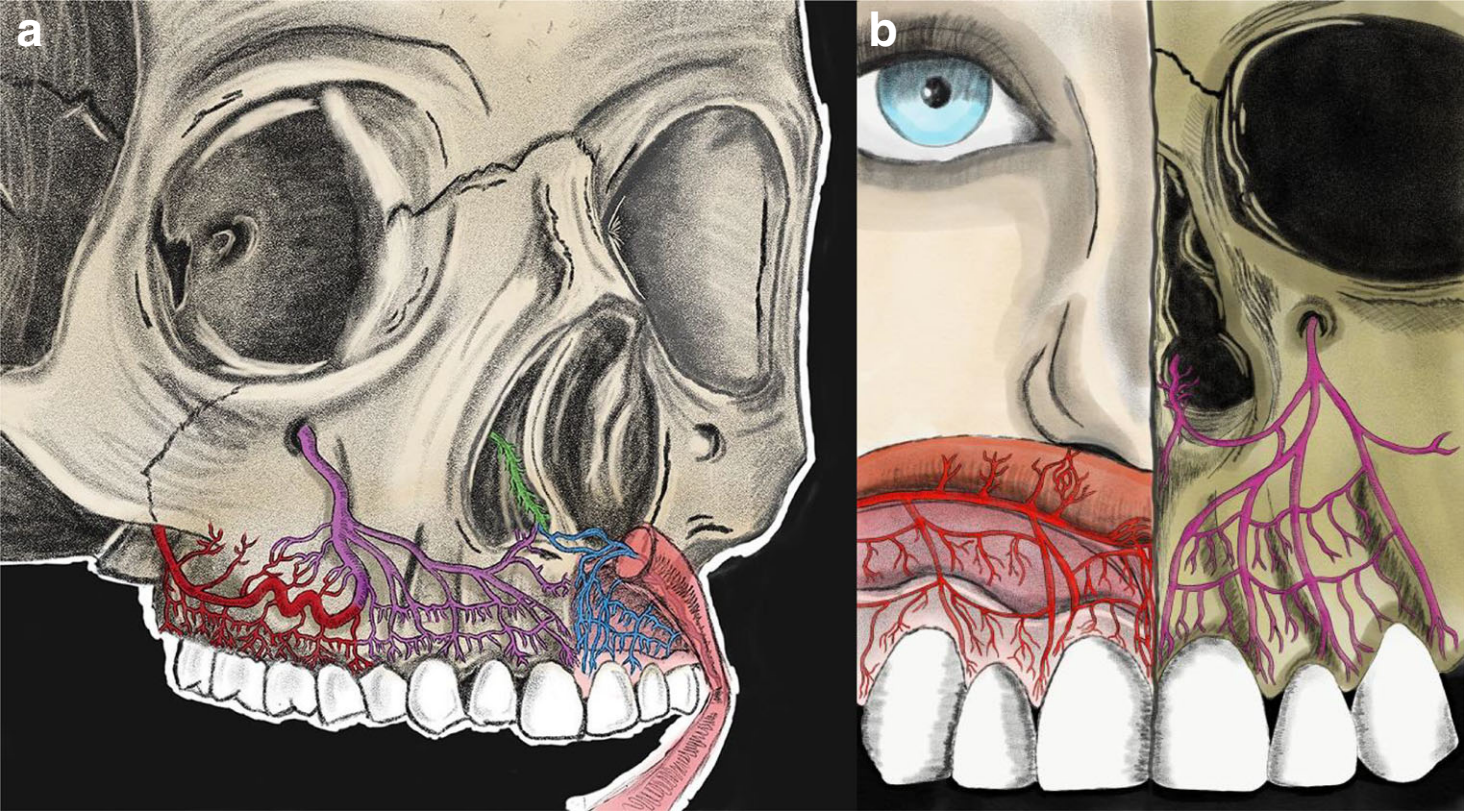
Schematic drawing of the vascular supply of the maxillary vestibule. **a** Overview of posterior superior alveolar artery (PSAA), infraorbital artery (IOA), and superior labial artery (SLA). **b** Esthetic zone blood supply, mucosa supply by SLA, and periosteal supply by IOA

Transverse periosteo-mucosal anastomoses were detected (Fig. 3). Also, besides these horizontal anastomoses, penetrating intraosseous branches was observed (Fig. 3). We were able to visualize the passage of arteries from the buccal side appearing on the interdental septum passing toward the palate. In the dentate ridges, on the surface of the mucosa, star-shaped terminal branches were detected, which were missing in the edentulous ridges (Fig. 5). The verdicts mentioned above have been compiled in Fig. 7, which describes the complexity of maxillary vestibule vascular supply.

## Discussion

In the current investigation, we were able to visualize previously undocumented periosteomucosal anastomoses, mucosal and periosteal vasculatures, and a network of anastomoses furnishing the maxillary vestibule with a focus on clinical relevance in regular dental practice. Latex milk injection and corrosion casting were successfully applied methods to detect the variations in the distribution of anterior and posterior vessels as well as of mucosal and periosteal vessels.

Numerous incision/flap designs have been introduced in implant-related and periodontal surgeries, to treat various diseases and to reconstruct hard tissue deficiencies by guided bone/tissue regeneration or autologous block grafting [1, 21–26, 36]. A proper flap design resulting in predictable wound healing is characterized by uninterrupted postoperative mucosal and periosteal microcirculation, which enables for protection of target tissues [37]. According to our results, some of the observed anatomical characteristics may contribute to clinical observations made in the daily practice related to postsurgical wound healing. The anterior segment of the maxillary vestibule displayed a vertical pattern of arterial pathways arriving from SLA in the superficial mucosal layer, targeting the mesial and distal papillae of the front teeth (Figs. 4, 5, 7). The papillae presented dense vascular supply, which is directly emanating from SLA. However, the gingival limbus blood supply is formed by branches that come from loop anastomoses between vertical branches (Fig. 4). If during implant-related surgeries vertical releasing incision is placed at the midline gingival zenith of anterior teeth, by damaging of these anastomoses, it might result in impaired blood supply which validates the frequent observation of sequels (gingival recessions, scars) [38]. Placement of vertical incisions in the esthetic zone represents a high risk of disturbed wound healing and consequent visible scar formation, with the exception of the upper labial frenulum. This is supported by our findings showing an abundance of arteries in that particular area (Figs. 4 and 5). Moreover, if scarring occurs, it would be hidden within the vertical fibers of the frenulum. Therefore, contrary to the concept of trapezoid flaps, horizontally extended flap elevation without vertical releasing incisions is preferable. If vertical releasing incisions are sequentially necessary for increased access and visibility during periodontal surgeries, implant placement, or augmentative procedures, L-shaped flaps with one vertical incision in the distal canine-first premolar region represent an optimal solution [21–25]. This approach is associated with relatively uninterrupted blood supply as well as minimal scarring for surgeries performed in the anterior and the posterior maxilla. The clinical concept of vertical incisions in the canine/first premolar area is supported by our outcomes showing only arteries with a reduced diameter in that particular region compared with the posterior major horizontal arteries.

Following flap elevation, horizontal releasing incisions or split-thickness dissection are frequently performed in the periosteum of the anterior maxilla. In case the surgical preparation is carried out with sharp instruments in the close vicinity of the piriform aperture, the risk of damaging the anastomosis between IOA and septal branches of the nasal cavity is high (Fig. 2). In such cases, postoperative epistaxis may occur. Additionally, in vascular CT analysis, the SLA presented ipsi- and contralateral anastomoses with the IOA which illuminates in case of any hazard of SLA or IOA, via these critical anastomoses the blood can circulate into the area (Fig. 6). In the posterior dentate maxilla, based on the horizontal pathway of mucosal and periosteal branches, vertical releasing incisions at the area of the second molar might block the pathway of the blood between distal and mesial tissues (Fig. 1). Moreover, increased intraoperative bleeding during sinus floor elevation or ridge augmentation might occur if distal vertical releasing incisions are placed, disrupting the distal blood supply of the posterior maxillary vestibule [11].

The star-like terminal branches observed in the most superficial layer were distributed over the entire vestibule, with increased density toward the labial frenulum (Fig. 5). The clinical observation of small localized hematomas following sub-mucosal injections might be explained by the occasional unintentional rupture of the star-shaped terminal arteries, resulting in postoperative discomfort. The density of the star-shaped terminal branches was enormously high in the dentate corpses compared with the edentulous, which might be explained by the fact that due to resorption of the connective tissue, these vessels are disappearing in edentulous ridges.

Transversal connections between buccal and palatal periosteal arteries were also observed in the form of arteries bridging the interalveolar bony septa in the papillary regions, which should be taken into consideration during immediate implant placement and periodontal surgeries. We found several transversal anastomoses that interconnected the blood supply of mucosal and periosteal layers both in the anterior and posterior zones (Fig. 3). According to our observations, the diameter of these transversal branches is inferior compared with horizontal, vertical mucosal and periosteal arteries. This implicates that careful split-thickness preparation might be feasible to achieve extended flap mobilization without severely disrupting periosteal blood supply [21–26]. Contrary to full-thickness mucoperiosteal flaps, this might result in less pronounced intraoperative bleeding and favorable wound healing due to minimally compromised postoperative blood flow, as suggested by several authors. Furthermore, surgical techniques in periodontal plastic surgery utilizing split-thickness dissection techniques are usually associated with minimal swelling, hematoma, and postoperative patient discomfort [21–26, 39].

## Conclusions

The presented macroscopic and radiographic cadaver survey successfully revealed the anatomical background behind several well-documented clinical phenomena related to periodontal and implant surgeries. Visualization of previously undocumented periosteomucosal anastomoses, mucosal vasculature, and a network of anastomoses furnishing the maxillary vestibule may enable clinicians to design less invasive flaps by avoiding or carefully placing vertical releasing incisions in critical anatomic regions. Furthermore, our observations strongly point to the significance of an undamaged periosteal layer to prevent compromised flap revascularization and wound healing disturbances in the maxillary vestibule.
